# Aldose Reductase and the Polyol Pathway in Schwann Cells: Old and New Problems

**DOI:** 10.3390/ijms22031031

**Published:** 2021-01-21

**Authors:** Naoko Niimi, Hideji Yako, Shizuka Takaku, Sookja K. Chung, Kazunori Sango

**Affiliations:** 1 Diabetic Neuropathy Project, Tokyo Metropolitan Institute of Medical Science, Tokyo 156-8506, Japan; yako-hd@igakuken.or.jp (H.Y.); takaku-sz@igakuken.or.jp (S.T.); sango-kz@igakuken.or.jp (K.S.); 2 Medical Faculty, Macau University of Science and Technology, Macau 999078, China; skchung@must.edu.mo

**Keywords:** Schwann cells, aldose reductase, polyol pathway, hyperglycemic conditions, immortalized Schwann cell lines, aldehyde detoxification

## Abstract

Aldose reductase (AR) is a member of the reduced nicotinamide adenosine dinucleotide phosphate (NADPH)-dependent aldo-keto reductase superfamily. It is also the rate-limiting enzyme of the polyol pathway, catalyzing the conversion of glucose to sorbitol, which is subsequently converted to fructose by sorbitol dehydrogenase. AR is highly expressed by Schwann cells in the peripheral nervous system (PNS). The excess glucose flux through AR of the polyol pathway under hyperglycemic conditions has been suggested to play a critical role in the development and progression of diabetic peripheral neuropathy (DPN). Despite the intensive basic and clinical studies over the past four decades, the significance of AR over-activation as the pathogenic mechanism of DPN remains to be elucidated. Moreover, the expected efficacy of some AR inhibitors in patients with DPN has been unsatisfactory, which prompted us to further investigate and review the understanding of the physiological and pathological roles of AR in the PNS. Particularly, the investigation of AR and the polyol pathway using immortalized Schwann cells established from normal and AR-deficient mice could shed light on the causal relationship between the metabolic abnormalities of Schwann cells and discordance of axon-Schwann cell interplay in DPN, and led to the development of better therapeutic strategies against DPN.

## 1. Introduction

**Figure 1:**
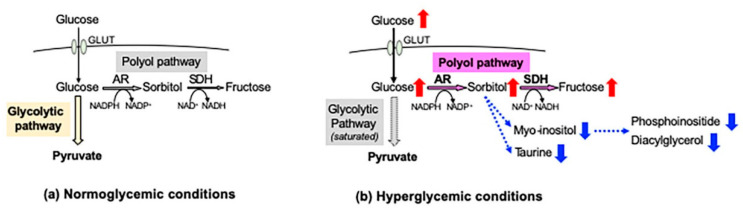
Glucose metabolism and the polyol pathway in the peripheral nervous system. Under normoglycemic conditions (**a**), most of the glucose incorporated into the cells via glucose transporters (GLUT) is metabolized into pyruvate through the glycolytic pathway, and less than 3% of the glucose enters the polyol pathway. However, under hyperglycemic conditions (**b**), the escalation of glucose flux into the polyol pathway results in the accumulation of sorbitol and fructose and the consumption of cofactors of AR (NADPH) and SDH (NAD^+^). Increased sorbitol contents lead to the depletion of myo-inositol and taurine, and reduced myo-inositol contents can be a cause of decreases in phosphoinositide and diacylglycerol. Red and blue arrows indicate increases and decreases in the amount of molecules, respectively.

In the peripheral nervous system (PNS), blood glucose is incorporated into cells via glucose transporters (GLUT 1 and GLUT 3) in an insulin-independent manner. Under normoglycemic conditions, most of the cellular glucose is converted to glucose 6-phosphate by hexokinase and further metabolized into pyruvate through the glycolytic pathway. However, under hyperglycemic conditions, the saturation of the glycolytic pathway escalates the flux of glucose through the polyol pathway, the first and major collateral route of glucose metabolism. The polyol pathway, which initially identifies in the seminal vesicle [1], consists of the reduction step of glucose to sorbitol and the oxidation step of sorbitol to fructose: the first reduction step is catalyzed by aldose reductase (AR) in a reduced nicotinamide adenosine dinucleotide phosphate (NADPH)-dependent manner, and the second oxidation step is catalyzed by sorbitol dehydrogenase (SDH) in an NAD-dependent manner, respectively (Figure 1). Polyol pathway hyperactivity induces multiple metabolic imbalances, described in the following sections, and is thereby considered as a major cause of diabetic peripheral neuropathy (DPN) [2], as well as other complications [3,4]. AR, being the rate-limiting enzyme of the polyol pathway, has been studied intensively as a target protein for the prevention and treatment of DPN. Although numerous AR inhibitors (ARI) have been developed with promising results in the cell and animal models of DPN, most of the clinical trials using them ended in failure due to the adverse effects and/or limited drug potency [5]. Currently, epalrestat (Table 1) is the only ARI available for clinical use in Japan, but its use is limited to the patients with DPN at early stages and stable glycemic control [6]. Despite the intensive studies over the past four decades, the significance of AR and the polyol pathway in the pathogenesis of DPN and effective therapies against it remains unknown.

AR is predominantly localized at Schwann cells in the PNS [7], and its activation and subsequent sorbitol accumulation in Schwann cells under hyperglycemic conditions appear to play a critical role in the development and progression of DPN [8]. Histopathological studies on peripheral nerve specimens from patients and experimental animals with DPN indicated the vulnerability of Schwann cells and its close association with nerve dysfunction, such as reduced nerve conduction velocity (NCV), decreased sensation, spontaneous pain, and impaired axonal regeneration [9]. In agreement with these findings, transgenic mice overexpressing human AR in Schwann cells displayed more severe neurological abnormalities than non-transgenic littermate mice under hyperglycemic conditions [10]. In contrast, AR-deficient mice created by gene manipulation showed a defense against neurological manifestations upon inducing diabetes [11]. However, the precise causal relationship between the AR/polyol pathway in Schwann cells and the pathogenesis of DPN remains unclear. Recent studies using AR-deficient mice [12] and Schwann cells [13] have raised new issues, such as crosstalk among the polyol and other collateral glucose-utilizing pathways. In this paper, we overview the previous studies regarding the roles of AR in Schwann cells under diabetic and non-diabetic conditions, and then we introduce immortalized Schwann cells, established in our laboratory [13,14,15,16], as valuable tools for the study of DPN, focusing particularly on AR and the polyol pathway.

## 2. Physiological and Pathological Roles of AR in Schwann Cells

### 2.1. Possible Functions of AR in Schwann Cells under Non-Diabetic Conditions

The aldo-keto reductase (AKR) superfamily consists of 15 family groups (AKR1-15) with various kinds of NADP(H)-dependent oxidoreductases [17]. AR belongs to the AKR1B subgroup of the AKR1 family (human and murine AR are termed as AKR1B1 and AKR1B3, respectively), and the subgroup is characterized by reducing saccharides and aldehydes derived from lipid peroxidation, steroids, and their derivatives [18]. As compared with other AKR1B constituents, AR seems to be more ubiquitously expressed [19,20,21,22], with predominant localization at the cytoplasm of the target tissues of diabetic complications, such as the vasculature, PNS (particularly Schwann cells), eyes, and kidneys [23]. AR has been receiving much attention as a possible culprit of DPN and other complications over the past four decades, whereas its physiological roles remain largely unknown. AR seems to be involved in the maintenance of cellular homeostasis through osmotic regulation and detoxification.

#### 2.1.1. Responses to Hyperosmotic Stress

In the kidney, hyperosmotic stress increases the AR activity and the amount of sorbitol, which balances the osmotic pressure of extracellular sodium chloride (NaCl) [24]. In addition to the osmoregulation, AR may be essential for the maturation of the urine concentrating mechanism [25,26]. The augmented flux into the polyol pathway under exposure to hypertonic conditions has been shown in many other cells, including Schwann cells [27,28,29]; however, the PNS is usually unaffected by extracellular osmotic stress and the osmoregulatory role of the AR/polyol pathway in Schwann cells remains obscure.

#### 2.1.2. Aldehyde Detoxification

AR and other AKRs are involved in the detoxification of reactive biogenic aldehydes, such as 4-hydroxy-2-nonenal (4HNE), acrolein, methylglyoxal (MG), and 3-deoxyglucosone (3-DG) [30]. Although AR has been suggested as an efficient catalyst for the reduction of these substances [31,32], neither AR gene ablation nor ARI treatment augmented their toxicity against Schwann cells [13,33]. These findings suggest the complex metabolic disposal system of reactive aldehydes, which will be fully discussed in Section 4.

#### 2.1.3. Steroid Metabolism

Neuroactive steroids, such as progesterone, testosterone and their reduced metabolites, have been shown to promote Schwann cell proliferation and myelin protein synthesis [34] and restore DPN through regulating myelin lipid profiles [35]. Schwann cells not only uptake these steroids via steroid receptors, but also produce and metabolize them [34]. AR is recognized to catalyze the reduction of progesterone and its related steroids [4], but whether their metabolism is affected by the polyol pathway hyperactivity under diabetic conditions or AR inhibition remains to be determined [36].

### 2.2. The Polyol Pathway as a Major Pathogenic Factor of DPN

Current evidence suggests that polyol pathway hyperactivity in the PNS (especially Schwann cells) under diabetic conditions triggers multiple metabolic abnormalities associated with DPN.

#### 2.2.1. Increased Sorbitol Contents

Because sorbitol is a membrane-impermeable substance, its accumulation was thought to impose osmotic stress on Schwann cells and the PNS (“Osmotic Hypothesis”). However, sorbitol concentrations under hyperglycemic conditions were found to be osmotically irrelevant, and the extracellular sorbitol level was significantly increased as compared with that under normoglycemic conditions in primary cultured Schwann cells [28]. These findings suggest that sorbitol can be released from Schwann cells by an unidentified transport mechanism [37], and the sorbitol-induced osmotic stress is unlikely to perturb Schwann cell function [38]. Rather, the increase in sorbitol contents leads to the depletion of other osmolytes, such as myo-inositol and taurine [39], whose changes may be more relevant to the pathogenesis of DPN (Figure 1). Because myo-inositol is an important constituent of the phospholipids, its depletion leads to a decrease in phosphoinositide and diacylglycerol contents, which, in turn, diminishes the activity of protein kinase C (PKC) and Na^+^–K^+^-ATPase [40,41,42]. Taurine works as an antioxidant, calcium modulator, and neurotransmitter, and its decrease has been suggested to cause oxidative and nitrosative stress in Schwann cells [43]. Although no studies addressed the efficacy of taurine in patients with DPN, its neuropharmacological potential is attracting attention [44].

#### 2.2.2. Redox State Changes

**Figure 2:**
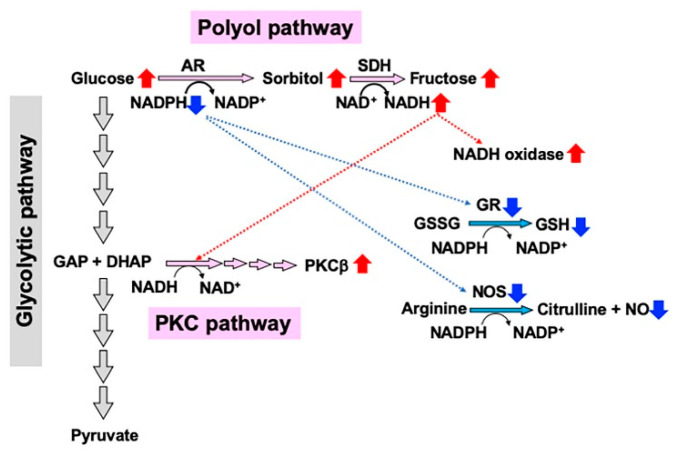
Redox state changes induced by the polyol pathway hyperactivity under high glucose conditions. Nicotinomide adenoside dinucleotide phosphate (NADPH) consumption during the first step catalyzed by aldose reductase (AR) suppresses the activities of glutathione reductase (GR) and nitric oxide synthase (NOS), which leads to the reduction in glutathione (GSH) and NO, respectively. NADH upregulation during the second step catalyzed by SDH increases the activities of NADH oxidase and PKCβ. GAP: glyceraldehyde 3-phosphate, DHAP: dihydroxyacetone phosphate. Red and blue arrows indicate increases and decreases in the amount or activity of molecules, respectively.

The polyol pathway hyperactivity and consequent consumption of cofactors of AR (NADPH) and SDH (NAD^+^) trigger a cascade of interrelated metabolic imbalances (Figure 2). NADPH is a common cofactor of AR, nitric oxide synthase (NOS), and glutathione reductase (GR), and its depletion due to the increase in AR activity leads to the inhibition of the other enzymes [45]. The reduced NOS activity and NO contents can be a cause of diminished nerve blood flow, whereas the decreases in reduced glutathione (GSH) by GR inhibition results in the excessive production of free radicals and the enhancement of oxidative stress [46]. During the second step of the pathway catalyzed by SDH, NAD^+^ is converted to the reduced form NADH. Because NADH is a substrate for NADH oxidase, the conversion leads to the upregulation of NADH oxidase activity and the production of superoxide anions, thereby contributing to oxidative stress [47]. Moreover, the NAD^+^/NADH redox state imbalances may be involved in the activation of PKCβ, which also plays a role in the development of DPN [2,48]. Regarding Schwann cells, however, direct evidence to prove these causal dependences is lacking.

#### 2.2.3. Formation of Dicarbonyl Compounds and Advanced Glycation Endproducts (AGEs)

As the second step of the polyol pathway, sorbitol is converted to fructose by SDH. Fructose is further metabolized into dicarbonyl compounds such as 3-deoxyglucose (3-DG) and methylglyoxal (MG), which are recognized as potent glycating agents and participate in the formation of AGEs [49,50]. Besides the AGEs induced Schwann cell injury and death [51,52], 3-DG and MG exhibit direct toxicity toward Schwann cells [33,53,54]. Recent studies suggest the formation of AGEs from exogenous (diet-derived) and endogenous (polyol-pathway-derived) fructose, and the fructose-derived AGEs have been attracting attention as a novel pathogenic factor for a variety of metabolic, inflammatory, and neurodegenerative diseases [55,56].

#### 2.2.4. PKC Activity Abnormalities

Hyperglycemic insults have been shown to affect PKC activity in a cell- and isozyme-specific manner; PKCα activity is reduced in Schwann cells, whereas PKCβ activity is increased in vascular endothelial and smooth muscle cells [2,57]. These findings led us to suppose the involvement of α isozyme inactivation in DPN and β isozyme activation in microangiopathy (including reduced nerve blood flow), respectively. AR/polyol pathway is suggested to interact with PKC signaling cascades and differentially influence the expression/activity of the α and β isozymes [48,58]. As described above, myo-inositol depletion can be a cause of the diminished α isozyme activity, whereas NAD^+^/NADH redox state imbalances appear to result in increased β isozyme activity. To support these ideas, treatment with ARI ameliorated the reduced PKCα activity in rat Schwannoma cells [42] and the enhanced PKCβ activity in endothelial and smooth muscle cells [59,60]. However, since the clinical trials of PKCβ inhibitors toward patients with DPN resulted in failure, research into PKC related to DPN, including its cross-talk with the AR/polyol pathway, has been on the decline.

#### 2.2.5. Epalrestat as a Pathogenesis-Based Medicine for DPN

The accumulating evidence indicates that AR and the polyol pathway are major culprits in the development and progression of DPN, and epalrestat, one of the AR inhibitors, has been developed and approved as a pathogenesis-based medicine in Japan (Table 1). However, its efficacy has been limited to the patients with mild DPN and stable glycemic control [6], and such unsatisfactory outcomes may result from multiple pathogenic factors for DPN, such as the collateral glycolysis pathways other than the polyol pathway [2,4,9,12], vascular abnormalities [61], and lifestyle-related factors other than hyperglycemia (e.g., obesity, dyslipidemia, hypertension, and smoking) [62]. The detailed understanding of the pathological roles of AR in DPN, particularly its relevance to other factors, may be required for developing more effective therapies. Immortalized Schwann cells established from normal and AR-deficient mice will be beneficial tools for the therapeutic approaches toward DPN, as discussed in the following sections.

## 3. IMS32 Schwann Cells as a Useful Tool to Study AR and the Polyol Pathway under Diabetic Conditions

### 3.1. IMS32 Cells Have Been Utilized for the Study of DPN

**Figure 3:**
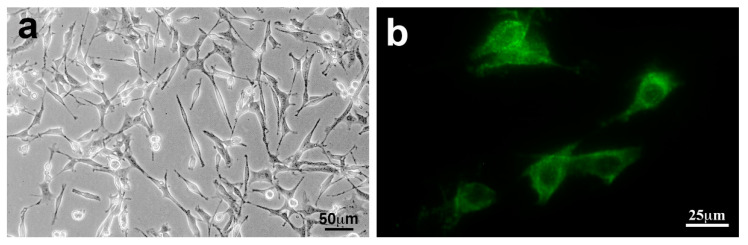
IMS32 cells showed distinct Schwann cell phenotypes such as spindle-shaped morphology under a phase-contrast microscope (**a**) and immunoreactivity to p75 low-affinity neurotrophin receptor (p75^NTR^) (**b**). Modified from Sango et al., *Experimental Diabetes Research* 374943 (2011) [16] (The publisher grants us the right to reuse the figure in other publications).

The culture system of Schwann cells is useful to investigate the high-glucose-induced alterations of mRNA/protein expression profiles and related metabolic pathways, including AR and the polyol pathway. Although the primary culture of rodent Schwann cells has been utilized for the study of DPN [63], a time-consuming process is needed to acquire good yields of Schwann cells with high purity. To avoid such an inconvenience, a variety of cell lines were established from Schwannoma cells and the long-term primary cultured Schwann cells by oncogene transfection or spontaneous immortalization [64]. Because of the high proliferative activity and uniformity, these cell lines are more suitable for molecular, biochemical, and metabolomic analyses than primary cultured Schwann cells. Conversely, the proliferative activity of the cell lines seems to conflict with the degree of differentiation and retention of Schwann cell property. A spontaneously immortalized mouse Schwann cell line IMS32 established from adult ICR mice [14] possesses both moderate growth potency and distinct Schwann cell phenotypes (e.g., spindle-shaped morphology, expression of glial cell markers, synthesis and secretion of neurotrophic factors and neuroprotective cytokines (Figure 3)). In addition, the IMS32 cell culture does not require any growth stimulants, necessary for the primary culture of Schwann cells (e.g., neuregulin-β and forskolin) [57]. Because of these advantages, IMS32 cells have been utilized for exploring the pathogenesis of DPN [12,15,33,65,66,67,68,69,70,71,72,73] (Table 2) and amyloid polyneuropathy [74], as well as the mechanisms of action of axonal regeneration-promoting molecules (e.g., ciliary neurotrophic factor [75], sonic hedgehog [76], and oxidized galectin-1 [77]). IMS32 cells are one of the best characterized Schwann cell lines and currently available for purchase at several companies. In contrast to these Schwann cell-like phenotypes, IMS32 cells differ from primary cultured Schwann cells in that the former are not contact-inhibited and that they form ball-shaped subcolonies when cultures reach confluence [14]. In addition, we have not succeeded in demonstrating that IMS32 cells myelinate neurites in co-culture with neurons. The higher proliferative activity of IMS32 cells compared with primary cultured Schwann cells might impede continuous and stable neuron-Schwann cell interplay, which usually take a month or longer to form the myelin structure. In mature peripheral nerves, Schwann cells play a key role in the conduction of nerve impulses, trophic support for neurons, production of extracellular matrix, regulation of immune responses, and axonal regeneration after injury [8,29]. Based on the findings, as described above, IMS32 cells appear to mimic some but not all characteristic features of naïve and activated Schwann cells.

### 3.2. IMS32 Cells Are Suitable for Exploring AR/Polyol Pathway-Related Abnormalities in DPN

In primary cultured Schwann cells [28] and a Schwannoma-derived cell line JS1 [27], AR expression and the polyol contents were unaltered in response to the hyperglycemic (20–30 mM) insults unless hyperosmotic stress (≥100 mM) was applied. In contrast, under exposure to high glucose (≥30 mM) conditions, we observed significant increases in AR mRNA/protein expression and the contents of sorbitol and fructose in IMS32 cells. Further, application of an AR inhibitor fidarestat (SNK-860, provided by Sanwa Kagaku Kenkyusho, Inabe, Japan) (Table 1) to the hyperglycemic milieu significantly diminished the polyol contents [15,33]. In agreement with our study, Cinti et al. (2015) [70] reported the high glucose-induced upregulation of AR activity and expression accompanied by enhanced lipid peroxidation and caspase 3 activity in IMS32 cells. These findings suggest that the culture of IMS32 cells under high-glucose conditions can be a useful in vitro model to study AR/polyol pathway-related abnormalities in DPN. Why the glucose concentrations corresponding to the plasma level in poorly controlled diabetic patients (20–30 mM) accelerate the polyol pathway in IMS32, but not in primary cultured Schwann cells or other Schwann cell lines, remains elusive. Sorbitol can be released from IMS32 cells into the culture media (Sango et al., unpublished data), but the cells might possess a much greater capacity than other Schwann cells to store sorbitol and other glucose-derived metabolites.

**Figure 4:**
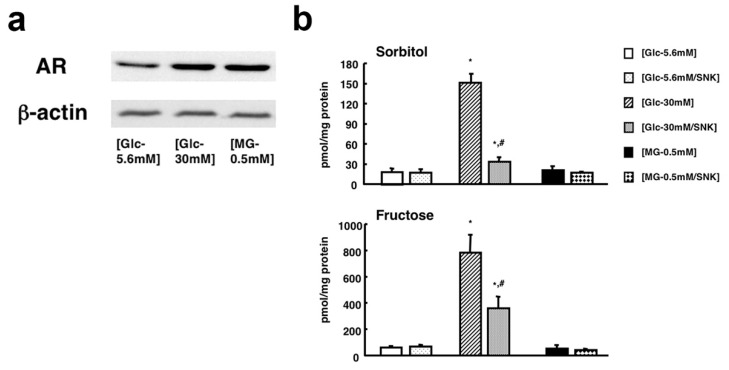
(**a**) High-glucose- and MG-induced upregulation of AR by Western blot analysis. (**b**) Intracellular contents of sorbitol (upper panel) and fructose (lower panel) after 7 days of exposure to (Glc-5.6mM), (Glc-30mM), and (MG-0.5mM) in the presence or absence of SNK-860 (1 μM). Values represent the mean + SEM of 6 experiments. * *p* < 0.01 as compared with (Glc-5.6mM) and # *p* < 0.01 as compared with (Glc-30mM). Modified from Sango et al., *Open Diabetes J.* **2008**, *1*, 1–11 [33] (The publisher grants us the right to reuse the figure in other publications).

The expression of AR in IMS32 cells was upregulated by exposure to MG (0.5 mM) under normoglycemic (5.6 mM) conditions, as well as exposure to hyperglycemic (30 mM) insults [33], as expected, since AR is known to be involved in the reduction of MG (cf. Section 2.1.2). However, the cellular reactions to MG were different from those to the high glucose. The MG-induced dose-dependent cell death was not associated with the increase in sorbitol and fructose contents. On the other hand, the high glucose insults led to the polyol pathway hyperactivity without a significant influence on the cell viability (Figure 4). These findings suggest that MG-induced upregulation of AR under normoglycemic conditions is a consequence of cytoprotective reactions, and the amount of glucose available for utilization through the polyol pathway appears to be insufficient to cause sorbitol accumulation. However, to prevent IMS32 cell death, the efficacy of AR for MG detoxification is insufficient.

According to the study by Hao et al. (2015) [69], primary cultured and IMS32 Schwann cells de-differentiated into immature cells (i.e., reduced expression of myelin proteins and enhanced expression of p75^NTR^, an immature Schwann cell marker) under hyperglycemic conditions via sorbitol accumulation. Treatment with epalrestat, an existing ARI used in Japan (Table 1) [6,78], ameliorated these changes. That study suggests the involvement of the polyol pathway in Schwann cell de-differentiation, which may be a cause of demyelination under diabetic conditions. However, ultrastructural studies of the peripheral nerves from patients with DPN at early stages showed axonal degeneration of myelinated and unmyelinated fibers without overt demyelination [79]. In addition, AR transgenic mice exhibited more severe neurological manifestations and nerve fiber atrophy than non-transgenic littermates when they were rendered diabetes by STZ, but no mice developed demyelination under that condition [80]. These findings suggest that the main pathology of DPN is axonal degeneration, which precedes demyelination observed in patients with DPN at advanced stages. Because axon−Schwann cell interplay is essential for the maintenance of peripheral nerve function, its discordance due to Schwann cell de-differentiation might affect both myelinated and unmyelinated fibers.

By employing IMS32 cells, we observed that novel ARIs such as fidarestat and ranirestat (AS-3201, provided by Sumitomo Dainippon Pharma Co., Ltd., Osaka, Japan) (Table 1) suppressed the high-glucose-induced upregulation of sorbitol and fructose contents more potently than epalrestat (Sango et al., unpublished data). Galactose is recognized the preferred substrate for AR, rather than glucose, and AR catalyzes the conversion of galactose to galactitol [81]. In our preliminary study, ranirestat suppressed galactose (25 mM)-induced upregulation of galactitol contents in IMS32 cells more effectively than epalrestat (Yako et al., in preparation). Despite the potent inhibitory effects against AR described above and the potential efficacy for patients with DPN, neither fidarestat nor ranirestat showed detectable benefits through the clinical trials [82].

## 4. Establishment of an AR-Deficient Schwann Cell Line IKARS1

### 4.1. Establishment and Characterization of IKARS1 Cells

**Figure 5:**
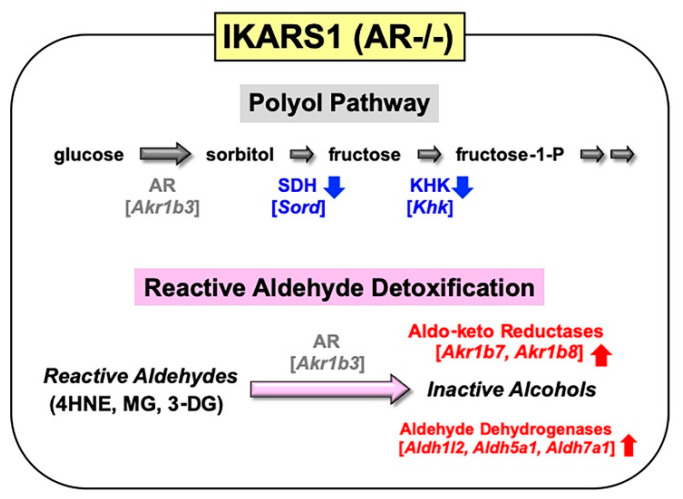
Establishment and characterization of IKARS1 cells. Marked down-regulation of polyol pathway-related enzymes (SDH, KHK) mRNA expression, and significant upregulation of aldo-keto reductases (AKR1B7, AKR1B8) and aldehyde dehydrogenases (ALDH1L2, ALDH5A1, ALDH7A1) mRNA expression in IKARS1 cells compared with those in 1970C3 cells. The upregulated enzymes might take over the AR detoxifying function. The genes encoding the respective enzymes are expressed as their abbreviations in square brackets.

Spontaneously immortalized Schwann cells were established not only from normal mice, but from murine disease models (e.g., Charcot–Marie-Tooth disease, neurofibromatosis and lysosomal storage diseases) [83]. These cell lines sufficiently represent the pathological features of the mutant mice, and they will be beneficial tools for studying the PNS lesions in the relevant diseases. In a similar manner, we obtained immortalized Schwann cell lines from AR-knockout C57BL/6 mice. Because our first trial to establish Schwann cell lines from wild-type littermates ended in failure, we employed one of the cell lines established from normal C57BL/6 mice in our institute as control of the AR-knockout cell line. These cell lines, termed IKARS1 (immortalized knockout AR Schwann cells 1) and 1970C3, respectively, showed distinct Schwann cell phenotypes similar to IMS32 cells, such as spindle-shaped morphology, expression of glial cell markers (S100β, p75 low-affinity neurotrophin receptor, etc.) and synthesis/secretion of neurotrophic factors [13]. Conditioned media collected from IKARS1 and 1970C3 cells enhanced neurite outgrowth from cultured adult mouse DRG neurons, and both cell lines secreted NGF and glial-cell-line-derived neurotrophic factor (GDNF) into the culture medium. The deficient AR activity in IKARS1 cells was confirmed by the galactose (25 mM)-induced increase in galactitol contents in 1970C3 cells but not in IKARS1 cells. A marked down-regulation of mRNA/protein expression of the enzymes downstream of AR in the polyol pathway (i.e., SDH and ketohexokinase (KHK)) in IKARS1 cells compared with 1970C3 cells (Figure 5) is consistent with the high-glucose-(30 mM)-induced increases in the sorbitol and fructose contents in 1970C3 cells but not IKARS1 cells. These findings indicate the absence of the polyol pathway in IKARS1 cells.

It is also noteworthy that mRNA expression of aldo-keto reductases (AKR1B7 and AKR1B8) and aldehyde dehydrogenases (ALDH1L2, ALDH5A1, and ALDH7A1) was significantly up-regulated in IKARS1 cells compared with 1970C3 cells (Figure 5). AKR1B7 and AKR1B8 are considered AR-related enzymes because of the high degree of sequence similarities to AR [52], and the AKRs and ALDHs that are up-regulated in IKARS1 cells appear to be involved in the reduction and oxidation of various aldehydes [84]. Although AR is suggested to be an efficient catalyst for the reduction in 4HNE, MG, and 3-DG (cf. Section 2.1.2), the cell viability assays revealed no significant differences in the relative survival ratios between IKARS1 and 1970C3 cells after exposure to these substances. The findings that AR gene deletion does not augment the aldehyde toxicity imply functional redundancies among AKRs and ALDHs regarding the metabolic disposal system of these aldehydes in Schwann cells. Because exposure to these aldehydes further up-regulated mRNA expression of AKR1B7 and AKR1B8, but not ALDHs, in IKARS1 cells, the reactive aldehyde detoxification can be taken over by AKR1B7 and AKR1B8 in the absence of AR.

### 4.2. Establishment of IWARS1 Cells and Future Studies with IKARS1 and IWARS1

The second trial to establish Schwann cell lines from wild-type littermates of AR-deficient C57BL/6 mice was successful. One of the cell lines termed IWARS1 (immortalized wild-type AR Schwann cells 1) possess distinct Schwann cell phenotypes similar to 1970C3 cells (Niimi et al., in preparation). Unlike IMS32 cells, neuregulin-β is required for the passage of IKARS1, 1970C3 and IWARS1 cells. The lower growth potency of these cells compared with IMS32 cells might be advantageous for myelination under co-culture with neurons, although we have not yet confirmed that they possess this capability.

**Figure 6:**
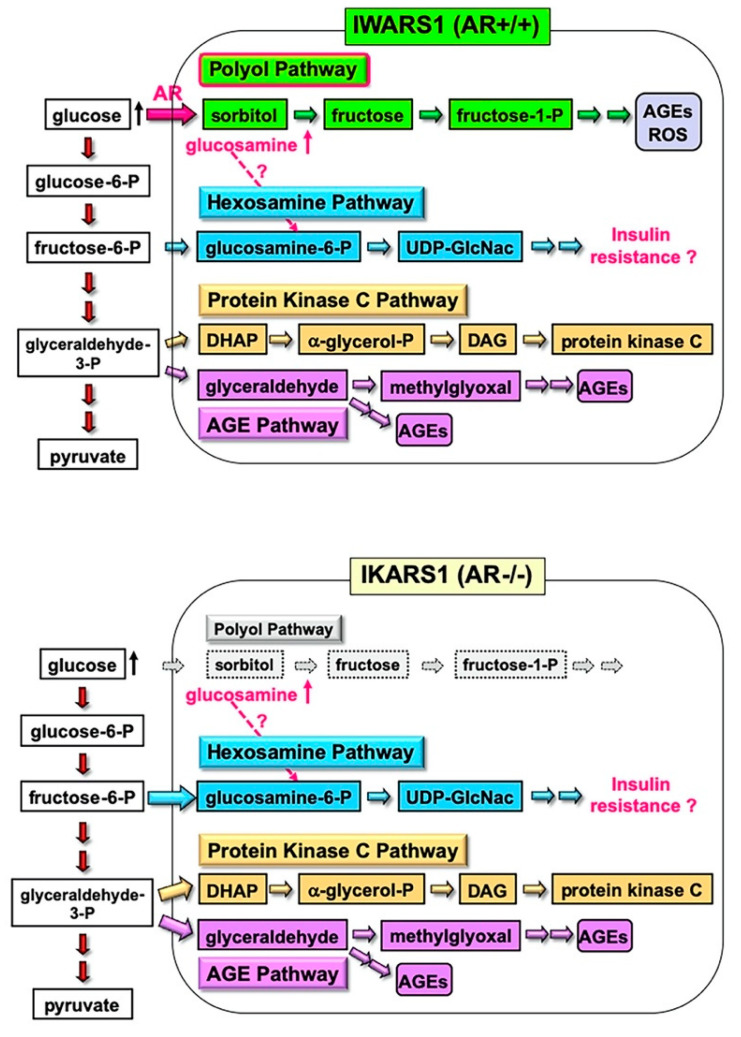
IWARS1 and IKARS1 cells established from wild-type and AR-deficient C57BL/6 mice as useful tools for exploring collateral glucose-utilizing pathways. Our current investigation using these cell lines focuses on the mechanisms of glucosamine accumulation in the PNS under hyperglycemic conditions regardless of the presence or absence of AR, and its association with hexosamine pathway hyperactivity that may impair insulin signaling.

The IWARS1 cells are “true controls” of IKARS1 cells, and these cell lines can be utilized to explore various collateral glucose-utilizing pathways (polyol pathway, hexosamine pathway, PKC pathway and AGE pathway) in relation to AR (Figure 6). The AR knockout mice, when rendered diabetes by streptozotocin, showed defense against neurological manifestations by 12 weeks after onset of diabetes [11]. However, Mizukami et al. (2020) [12] recently reported the reduced NCV in AR knockout mice with 16 weeks of diabetes, suggesting that other pathways, downstream or independent of the polyol pathway, are involved in the pathogenesis of DPN with long duration of hyperglycemia. They also observed an accumulation of glucosamine in the sciatic nerves of diabetic AR knockout and wild-type mice and direct toxicity of glucosamine toward cultured DRG neurons and IMS32 Schwann cells. While how glucosamine is up-regulated under diabetic conditions remains unclear, glucosamine can be metabolized to glucosamine-6-phosphate, constituents of the hexosamine pathway, the second collateral glycolysis pathway [2,4]. IKARS1 and IWARS1 cells will be essential reagents to determine the involvement of the AR and hexosamine pathway in the pathogenesis of DPN, as well as other pathogenic factors of DPN related to the polyol pathway, such as glycated, oxidative and nitrosative stress (cf. Section 2.2).

## 5. Conclusions

As we stated in the title of this paper, the involvement of AR and the polyol pathway in Schwann cell dysfunction under diabetic conditions and the pathogenesis of DPN is an old and new target for developing effective therapies against the diabetic complications, such as DPN. Despite growing evidence, as illustrated in Section 2, researchers studying DPN face the reality that much basic and clinical research work is still required. As described in Section 3, IMS32 cells under hyperglycemic conditions are a useful in vitro model to study the polyol-pathway-related abnormalities. The IKARS1 and IWARS1 cells introduced in Section 4 are expected to shed some light on the cross-talk between the polyol pathway and other pathogenic factors of DPN. We look forward to the exponential knowledge progression in the novel understanding of AR and related pathways in DPN using these Schwann cell lines through international collaborative efforts.

## Figures and Tables

**Table 1:** Aldose reductase inhibitors described in this article.

Name	Epalrestat (Kinedak^®^)	Fidarestat (SNK-860)	Ranirestat (AS-3201)
Molecular Formula	C_15_H_13_NO_3_S_2_	C_12_H_10_FN_3_O_4_	C_17_H_11_BrFN_3_O_4_
Molecular Weight	319.4	279.2	420.2
International Union of Pure and Applied Chemistry (IUPAC) Name	2-[(5Z)-5-[(E)-2-methyl-3-phenylprop-2-enylidene]-4-oxo-2-sulfanylidene-1,3-thiazolidin-3-yl]acetic acid	(2S,4S)-6-fluoro-2’,5’-dioxospiro[2 ,3-dihydrochromene-4,4’-imidazolidine]-2-carboxamide	(3R)-2’-[(4-bromo-2-fluorophenyl)methyl] spiro[pyrrolidine-3,4’-pyrrolo[1,2-a]pyrazine]-1’,2,3’,5-tetrone
Chemical Structure	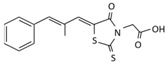	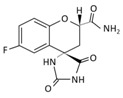	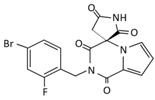
Current Status	It is commercially available in Japan	Its development was terminated	Its development was terminated

**Table 2:** IMS32 Schwann cells utilized for the study of diabetic peripheral neuropathy (review articles are not included).

References	Major Findings
Sango et al. (2006) [15]	High-glucose (≥30 mM) conditions increased AR mRNA/protein expression and the intracellular contents of sorbitol and fructose.
Ota et al. (2007) [65]	Metformin inhibited MG-induced apoptosis via JNK signaling pathway.
Sango et al. (2008) [33]	Both high-glucose and MG-induced upregulation of AR and oxidative stress markers (4-hydroxy-2-nonenal, acrolein and hexanoyl lysine).
Tosaki et al. (2008) [66]	Hyperglycemic insults inhibited nerve growth factor (NGF) secretion from IMS32 cells, being a cause of reduced neurite outgrowth activity of the conditioned media.
Kim et al. (2011) [67]	A mixture extract of *Dioscorea japonica* Thunb and *Dioscorea nipponica* Makino exerted neurite outgrowth-promoting activity on dorsal root ganglion (DRG) neurons, but not NGF induction effects on primary cultured and IMS32 Schwann cells.
Kim et al. (2013) [68]	Long-term (>8 wk) hyperglycemic insults up-regulated the expression of genes that promote glycolytic pathway and down-regulated the expression of genes involved in fatty acid metabolism, pentose–phosphate pathway and TCA cycle.
Hao et al. (2015) [69]	Hyperglycemic insults induced Schwann cell de-differentiation and suppressed insulin-like growth factor 1 expression via polyol pathway hyperactivity.
Cinci et al. (2015) [70]	Hyperglycemic insults enhanced AR expression, lipid peroxidation, and caspase-3 activity in a time-dependent manner (2 days < 7 days < 14 days).
Min et al. (2018) [71]	Human mobilized mononuclear cells (hMNC) restored DPN in STZ-mice and enhanced the expression of myelin protein zero in co-cultured IMS32 cells through hepatocyte growth factor-paracrine activity.
Tatsumi et al. (2019) [72]	Omega-3 polyunsaturated fatty acids alleviated oxidative stress-induced cell death by activating the antioxidant enzymes through the Nrf2 pathway.
Kato et al. (2019) [73]	Recurrent short-term hypoglycemic (2.5 mM) and hyperglycemic (25 mM) insults induced apoptosis and oxidative stress via the ER stress response.
Mizukami et al. (2020) [12]	Glucosamine induced IMS32 cell death via insulin signaling impairment and ATP depletion.

## Data Availability

Not applicable.

## Abbreviations

AKR	Aldo-keto reductases
ALDH	Aldehyde dehydrogenase
AR	Aldose reductase
3-DG	3-Deoxyglucosone
DPN	Diabetic peripheral neuropathy
DRG	Dorsal root ganglion
GDNF	Glial cell line-derived neurotrophic factor
4HNE	6-Hydroxy-2-nonenal
IKARS1	Immortalized knockout aldose reductase Schwann cells 1
IMS32	Immortalized mouse Schwann cells 32
IWARS1	Immortalized wild-type aldose reductase Schwann cells 1
KHK	Ketohexokinase
MG	Methylglyoxal
NADPH	Reduced nicotinamide adenosine dinucleotide phosphate
NGF	Nerve growth factor
p75^NTR^	p75 low-affinity neurotrophin receptor
PKC	Protein kinase C
PNS	Peripheral nervous system
SDH	Sorbitol dehydrogenase
